# Safety of Aesthetic Medicine Procedures in Patients with Autoimmune Thyroid Disease: A Literature Review

**DOI:** 10.3390/medicina58010030

**Published:** 2021-12-24

**Authors:** Kamil Adamczyk, Ewa Rusyan, Edward Franek

**Affiliations:** 1 Adamczyk Clinic, Żyzna 4, 03-613 Warsaw, Poland; kontakt@dradamczyk.pl; 2 Clinic of Anaesthesiology and Intensive Therapy, Central Clinical Hospital of the Ministry of Interior and Administration in Warsaw, Wołoska 137, 02-507 Warsaw, Poland; 3 Department of Conservative Dentistry, Warsaw Medical University, Żwirki I Wigury 61, 02-091 Warsaw, Poland; erusyan@wum.edu.pl; 4 Clinic of Internal Medicine, Endocrinology and Diabetology, Central Clinical Hospital of the Ministry of Interior and Administration in Warsaw, Wołoska 137, 02-507 Warsaw, Poland

**Keywords:** aesthetic medicine, autoimmune thyroid diseases

## Abstract

Autoimmune thyroid diseases are the most common organ-specific autoimmune diseases, affecting 2–5% of the world’s population. Due to the autoimmune background of thyroid diseases, we analyzed a wide range of cosmetic procedures, from minimally invasive cosmetic injections (mesotherapy) to highly invasive procedures, such as lifting threads. Out of the seven categories of treatments in aesthetic medicine analyzed by us—hyaluronic acid, botulinum toxin, autologous platelet-rich plasma, autologous fat grafting, lifting threads, IPL and laser treatment and mesotherapy—only two, mesotherapy and lifting threads, are not recommended. This is due to the lack of safety studies and the potential possibility of a higher frequency of side effects in patients with autoimmune thyroid diseases.

## 1. Introduction

Autoimmune diseases (AITD) of the thyroid gland—hypothyroidism and hyperthyroidism—mainly occur in areas without iodine deficiency, including in most wealthy countries [1], where aesthetic medicine procedures are still gaining in popularity. The exact pathogenesis of both autoimmune hypothyroidism and hyperthyroidism is unknown, but genetic factors play a role in both diseases [2]. Apart from genetic factors, AITD also seems to be influenced by epigenetic factors (the influence of IFNα on the expression of the thyroglobulin gene is postulated) [3] and environmental factors, such as iodine, smoking, medications, infection, or even stress [4]. Autoimmune thyroid diseases are the most common organ-specific autoimmune diseases, affecting 2–5% of the world’s population [5], and are more predominate in women than in men, with evidence of racial differences. Overall incidence of autoimmune hypothyroidism is 350/100,000/year in women and 80/100,000/year in men, and for autoimmune hyperthyroidism, the incidence is 80/100,000/year in women and 8/100,000/year in men [6]. The most common causes of autoimmune hypothyroidism and hyperthyroidism are Hashimoto thyroiditis (HT) and Graves’ disease (GD), respectively [7]. Patients with autoimmune thyroid diseases, in particular hypothyroidism, are a unique group of patients who declare a reduced quality of life and dissatisfaction with the medical services they receive [8]. Additionally, in patients with autoimmune hyperthyroidism, a reduction in the quality of life is observed, and mental health issues are more common than in the general population [9].

Cosmetic procedures mostly rely on the subcutaneous or intracutaneous injection of exogenous substances, but in recent years, treatments based on platelet-rich plasma and autologous tissue transplantation have become increasingly popular. A few studies have researched the safety of using minimally invasive cosmetic procedures in patients with autoimmune diseases [10]. Due to the autoimmune background of thyroid diseases, we analyzed a wide range of cosmetic procedures, from minimally invasive cosmetic injections (mesotherapy) to highly invasive procedures, such as lifting threads.

In the present article, we will analyze the current state of knowledge about the safety and effectiveness of the most popular methods of aesthetic medicine in the context of the coexistence of autoimmune thyroid diseases.

The search strategy involved searching electronic databases Medline, Pubmed, and Google Scholar for all articles on the topic of autoimmune thyroid diseases related to cosmetic procedures. The bibliographic references of the searched articles were examined for additional citations. The following keywords were used alone or in combination, when appropriate: “autoimmune thyroid disease”, “autoimmune disease”, “hashimoto”, “graves”, “hyaluronic acid injection”, “botulinum”, “botox”, “platelet rich plasma”, “PRP”, “conditioned plasma”, “fat grafting”, “fat transfer”, “fat injection, “lifting threads”, “PDO threads, ”biorevitalising threads”, “IPL”, “laser”, and “mesotherapy”.

## 2. Hyaluronic Acid

With age, the volume and elasticity of the tissues of the human body decreases, which is related to their dehydration. This process is especially visible on the face [11]. The main natural molecule responsible for binding water in the skin is hyaluronic acid (HA) [12].

The first commercially available tissue filler for aesthetic use was a product called Zyderm (Allergan, Dublin, Ireland), approved by the FDA in 1981. It was made of bovine collagen, which was associated with numerous allergic reactions [13]. The problems of allergic reactions were only solved after the introduction of the artificial hyaluronic acid molecule (Galderma S.A., Lausanne, Switzerland) on the market in 2003 [14]. According to the American Society of Plastic Surgeons, as of 2020, there had been over 3.4 million soft tissue fillers procedures in the USA [15], with over 160 products currently available on the market from over 50 different manufacturers [16]. Such a widespread use of tissue fillers is also associated with an increasing number of complications after hyaluronic acid procedures, which every practitioner of aesthetic medicine should be aware of. These include, among others, injection site reactions, such as edema, erythema or bruising, infection, skin discoloration, foreign body granulomas, or even tissue necrosis [16].

In one study, the authors assessed whether the administration of hyaluronic acid would affect the level of antibodies, such as thyroglobulin or anti-thyroid peroxidase, on the Iraqi female population. The study showed no differences with the control group, both in terms of the thyroid and other antibodies screened [17]. The use of hyaluronic acid in the course of other autoimmune diseases, such as scleroderma [18] or systemic sclerosis, has also been described, without side effects of the treatment [19].

Tissue fillers are widely used all over the world; however, in some patients, nodular masses, which are palpable, unintended hyaluronic acid accumulations, may be observed at the injection sites, which can result in patient dissatisfaction. These soft tissue augmentations can be divided into noninflammatory and inflammatory nodules. The former usually result from bad technique, when too much material is injected in one area. Inflammatory nodes can be caused by infection or by foreign body granulomas—the latter occur primarily in patients with autoimmune diseases. The exact frequency of this type of disorder in people with autoimmune diseases is unknown; however, it occurs more often in the population of people with autoimmune diseases [20] than in the general population, with inflammatory skin reactions estimated at approximately 0.42% [21]. It is still unknown whether this reaction is caused by hyaluronic acid or the synthetic hyaluronic acid additives [22]. Special attention should be paid to areas with high mobility, such as lips, which are at the highest risk of lump formation, even in healthy patients [16]. In the opinion of the authors, autoimmune diseases should not be an absolute contraindication to the use of fillers, as it is stated in some sources [23]. Practitioners should, however, be knowledgeable and clear about the increased risks of complications after hyaluronic acid procedures in those patients.

## 3. Botulinum Toxin

Botulinum toxin injections are one of the most popular treatments in aesthetic medicine, with an annual number of approximately 3 million procedures, per year, worldwide [24]. Currently, botulinum toxin is most used in aesthetic medicine; it is one of the most effective substances in the fight against wrinkles, allowing patients to achieve visible and natural effects. In Poland, the use of botulinum toxin A and B is allowed, but the use of type A is much more widespread. Apart from its use in dermatology and aesthetic medicine, botulinum toxin is also used in ophthalmology for strabismus or blepharospasm treatment, neurology to treat spasticity, contractures, and migraines, and in urology in the treatment of overactive bladder syndrome.

In vitro studies by Gregoric et al. indicated a molecular similarity between the botulinum toxin type A molecule and thyroid autoantigens [25], which, theoretically, could trigger an immune reaction after the administration of botulinum toxin in a patient with Hashimoto’s disease. As authors described, some antibodies produced after the botulinum toxin injection (anti-Btx) can bind into the thyrotropin receptor (TSH-R). This can therefore result in induction of anti-TSH-R antibodies that inhibit TSH-R signaling (TSH-R-blocking antibodies—TSHR-Bab), leading to elevated levels of TSH in the blood. Apart from this single scientific report, the authors of the study did not identify any other works supporting the thesis about the influence of botulinum toxin on thyroid function.

Nonetheless, botulinum toxin is used to treat eye complication in thyroid eye diseases, such as strabismus [26] or upper eyelid retraction [27,28,29,30]. Apart from the effectiveness of ophthalmic treatment with botulinum toxin, its safety is also emphasized, even in the acute inflammatory phase of the disease [30]. The producers of botulinum toxin type A agents available in Poland do not indicate in the summary of product characteristics that autoimmune diseases are a contraindication to the use of the product.

## 4. Autologous Platelet-Rich Plasma

Platelet-rich plasma has been used in medicine for years due to the increased content of platelets and growth factors, such as epidermal growth factor (EGF), insulin growth factor (IGF 1), platelet-derived growth factor (PDGF-AA, -AB and -BB), transforming growth factor (TGF-ß1 and -ß2), and vascular endothelial growth factor (VEGF A and C), [31] that stimulate, among others, fibroblasts, adipocytes, and endothelial cells. By stimulating these cells, regenerative processes are stimulated, hence its wide application in healing wounds or treating joints.

Treatments with PRP are gaining popularity. Increasingly, these procedures are used in the treatment of various diseases on the border of different specialties [32,33,34,35,36,37,38]. Apart from aesthetic medicine and plastic surgery, PRP is particularly popular in orthopedics. Prospective double-blind clinical trials comparing the use of PRP and hyaluronic acid in osteoarthritis indicate superiority of PRP treatment due to the positive local anti-inflammatory effects [39]. Another prospective randomized clinical trial showed better results from the use of PRP than steroids in osteoarthritis. Animal studies have shown a positive effect of treating rheumatoid arthritis with PRP [40], as have human trials [41]. Research on the use of PRP in the treatment of neurological diseases, in particular multiple sclerosis, also appears promising on animal models [42]. Additionally, there are conferences reports about the use of intra-thyroid injections of platelet-rich plasma in the treatment of hypothyroidism. In one of them, the authors indicate that this type of therapy reduces the value of TSH and anti-TG antibodies [43].

However, it should be remembered that thyroid diseases negatively affect the function of platelets, the functioning of which depends on the effectiveness of this treatment. In people with thyroid diseases, an increase in bleeding time and blood coagulation factors, such as APTT (activated partial thromboplastin time) or ACT (activated recalcification time), is observed [44]. Thyroid hormones act mainly on receptors located in the cell nucleus. Platelets do not have a nucleus, but the effect of thyroid hormones on the cytoplasmic elements of the cell is known [45]. Moreover, it is postulated that the thyroid hormones influence megakaryocytes—nucleated stem cells from which platelets are formed. In addition, an increased number of megakaryocytes in the bone marrow has been observed in patients with hypothyroidism [46]. Thyroid diseases affect mean platelet volume (MPV) and platelet distribution width (PDW). These measurements are elevated in Hashimoto thyroiditis [47], hyperthyroidism [48], and subclinical hypothyroidism [49,50,51,52]. Younger forms of platelets usually result in higher MPV [53]. The effect of serum T4 levels on the number of platelets has also been proven—an increased amount of T4 corresponds to increased platelets counts [54]. The same study did not show any effect of thyroid hormones on MPV. On the other hand, studies demonstrating alternated MPV in the course of thyroid diseases showed no change in platelet count [47,48,49,50,51,52].

Masunga et al. conducted a study in which they analyzed platelet-rich plasma separated from blood samples obtained from patients with Graves’ disease, Hashimoto’s thyroiditis, and idiopathic primary hypothyroidism [55]. Platelet aggregation in patients with untreated primary hypothyroidism was increased, and in those with untreated Graves’ disease, it was decreased. Since platelet aggregation is a direct measurement of platelet activation and its delivery autologous growth factors and cytokines into tissues, it can be assumed that the effectiveness of PRP treatments in hyperthyroidism will be reduced, while in hypothyroidism, it should not be changed. These considerations, however, require testing in a clinical setting. Furthermore, in the case of extensive procedures with the use of PRP, it is advisable to control the coagulation parameters in patients with thyroid disorders due to the increased risk of bleeding.

## 5. Autologous Fat Grafting

**Figure 1:**
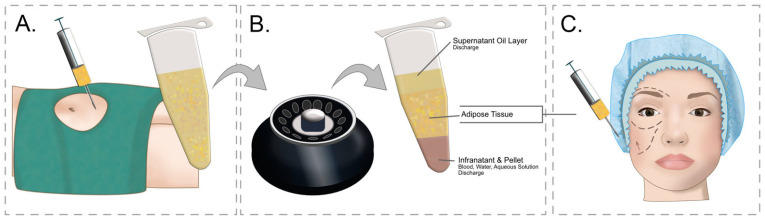
Study flow diagram and post hoc derivative prediction models: screening test and diagnostic test results. (**A**) Adipose tissue is collected from the recipient site—most often the abdomen or thighs. After the skin is anesthetized with a local anesthetic, an incision is made in the skin through which a syringe with a cannula is inserted and Klein’s solution is administered subcutaneously, which anesthetizes the tissues and causes vasoconstriction, minimizing surgical bleeding. After the tissues are anesthetized, a cannula with an attached syringe is inserted through the opening in the skin, in which a vacuum is created. The sliding movements of the cannula detach and suck off the fat. (**B**) After collecting the fat, it is centrifuged in order to separate it from other fractions. The centrifuged fat is then crushed by passing it through special narrowed connectors. (**C**) After the fat is prepared, it is cannulated into the regions where fat loss occurs.

Adipose tissue transplants have been performed for a long time, but only the groundbreaking work of Coleman [56,57] brought about widespread understanding and development of this technique. Currently, it is a minimally invasive procedure performed on an outpatient basis. Depending on the treated area, between 20–60 mL of adipose tissue is collected, in the case of treatment of small areas, e.g., ulcers or face volumetry, and up to several liters of adipose tissue is collected in the case of buttock or breast augmentation. The procedure of fat tissue extraction (liposuction) is performed under general or local anesthesia. As a rule, procedures collecting larger amounts of adipose tissue are performed under general anesthesia. However, the development of modern liposuction techniques [58] and the introduction of tumescent anesthesia by Klein [59] now allow for practically any procedure to be performed without the need for sedation. The collected fat is then centrifuged to separate the fluid fraction from the fat, then the separated fat is passed through sieves to get rid of lumps before being fed to the tissues. This process is shown in Figure 1.

Autologous fat transfer is currently one of the most popular plastic surgery procedures, and its enormous popularity is evidenced by the fact that, in a recent survey, over 80% of plastic surgeons admitted that they had performed such a procedure [60]. The recent identification of fat graft component stem cells has increased the value of the procedure from purely aesthetic to one with regenerative potential [61]. Autologous fat is considered a safe and biocompatible material [62]. Currently, apart from cosmetic procedures, autologous fat transplantation is used in the treatment of some conditions, including autoimmune diseases, e.g., scleroderma [63,64,65] or systemic sclerosis [66].

There have been reports of a granulomatous reactions to the administration of autologous fat transplant [67]; however, it seems to be the result of poor surgical techniques [68] and not of the patient’s comorbidities. Nevertheless, it seems that autologous fat transplantation procedure is safe in patients with autoimmune thyroid disease; however, its effectiveness in terms of graft survival may be reduced in this group of patients with autoimmune disease [69,70,71].

## 6. Lifting Threads

**Figure 2:**
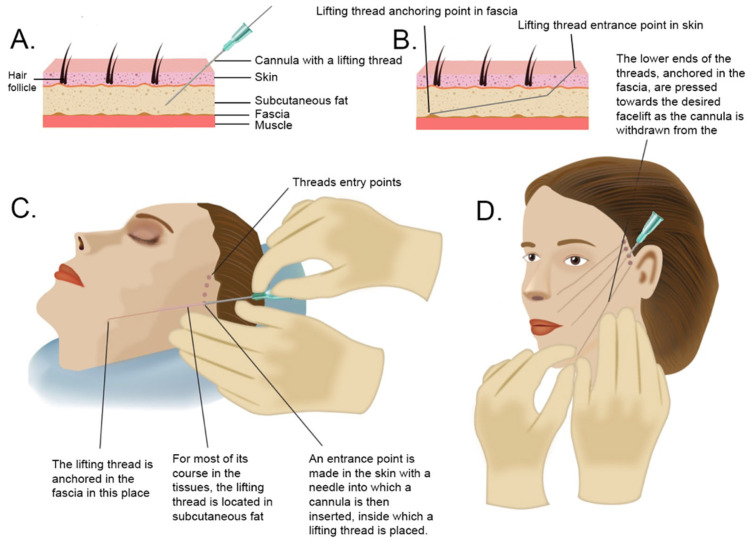
Study flow diagram and post hoc derivative prediction models: screening test and diagnostic test results. (**A**) Cross-section through the skin and deeper tissues. After making a hole in the skin with a needle, a cannula with a lifting thread inside is inserted into the subcutaneous fat layer. (**B**) Diagram illustrating the technique of thread anchoring in the fascia. (**C**) In order to lift the middle part of the face, the threads are inserted in the area of the temples and in the subcutaneous fat layer led to the area of the cheek, mouth, and jaw line to anchor the thread in the fascia. (**D**) After inserting the cannula into the subcutaneous fat layer, with twisting movements, the cannula is placed in the place of a decent lift and then directed to the deeper layers of fat on the border with the fascia to anchor the thread in it. Then, the lift target point is pressed by the hand, the thread unlocks, and the cannula advances. The lifting thread remains in the tissues.

Lifting threads are specially modified surgical sutures made of absorbable materials, such as polydioxanone or polylactic acid. Polydioxanone can persist in tissues for up to 12 months, but is typically degraded by hydrolysis within 10–12 weeks [72], the median half-life of polyamic acid is 30 weeks [73]. The method of using lifting threads and the technique of their implantation were developed in 1996 by Dr. Marlen Sulamanidze [74]. The novelty of this technique was to create special threads that do not need to be sewn to the tissues to achieve a lifting effect. These threads have micro-spikes that anchor themselves in the tissues. Additionally, a system of cannulas, i.e., blunt needles, has been developed, thanks to which, lifting threads can be atraumatically inserted into the tissues. The procedure of facelifting using thread is shown in Figure 2.

Procedures using lifting threads, especially in the area of the face and neck, are becoming progressively popular due to their reduced invasiveness compared with surgical procedures; nevertheless, many plastic surgeons remain skeptical of these procedures [75]. It seems, however, that this type of procedure is a much less invasive alternative for patients considering facelift surgery. With the increasing popularity, there are reports of complications related to the use of lifting threads [76,77,78]; nonetheless, the procedure appears to be a relatively safe [79].

The authors did not identify any study that assessed the safety of using lifting threads in patients with autoimmune diseases. Some authors choose not to perform this type of procedure on patients with any autoimmune disease [79]. Additionally, Aptos, one of the largest producers of lifting threads, mentions autoimmune diseases as a contraindication to the procedure [80].

The materials from which the lifting threads are made seem to be neutral in terms of triggering autoimmune reactions and some of them are even used in the treatment of autoimmune diseases by blocking autoimmune systemic response [81,82]. Autoimmune diseases, however, are a relative contraindication—the safety of the thread has not been established in these patients. Surgical suture hypersensitivity is rare, but its incidence increases in autoimmune diseases [83]. Since lifting threads are made from the same materials as surgical sutures, the issue of hypersensitivity to lifting threads is probably similar.

Since the manufacturers of lifting threads show autoimmune diseases as contraindications to the use of the product, the authors of the study do not recommend their use in autoimmune diseases of the thyroid gland.

## 7. IPL and Laser Treatment

Laser therapy has been used for many years in various fields of medicine. Recently, the use of lasers and intense pulsed light (IPL) in dermatology and aesthetic medicine has gained enormous popularity. The laser therapy consists of emitting a beam of light of a certain intensity on a selected part of the body for a specified period of time. The lasers used in this type of treatment can send the beams in a continuous or pulsed manner. IPL also uses light to work, but unlike a laser, it consists of a broad spectrum of light, similar to incandescent light, which can affect larger areas of the skin.

Several types of lasers are used in dermatology, categorized according to the type of active medium responsible for producing the laser beam. Thus, there are solid-state lasers, where a crystal matrix is used with the ingredient of metal ions that absorb and re-emit photons, such as neodymium-YAG (Nd: Yag, used, e.g., to close dilated blood vessels or remove small nodules), erbium-YAG (Er: Yag, used in skin photo rejuvenation treatments), and ruby laser (useful for removing tattoos and discoloration). Gas lasers are another category, in which the crystal has been replaced with gas, which includes the immensely popular CO_2_ laser (used to remove scars, skin lesions, wrinkles), the argon laser (used to remove skin discoloration, treatment of rosacea), or the helium–neon laser (acne treatment). Gas lasers use dyes that fluoresce or dye solutions that do not fluoresce. These include a continuous dye laser and a pulsed dye laser used for facial treatment. Finally, semiconductor (diode) lasers are used for epilation.

The authors did not identify any studies showing a negative effect of dermatological laser therapy or IPL treatment on patients with autoimmune thyroid diseases. Besides, some types of lasers therapies called low-level laser therapies (LLLT) appear to have a positive effect on the treatment of autoimmune diseases of the thyroid gland [84,85]. Moreover, there are scientific reports indicating the positive effect of laser and IPL on patients with autoimmune connective tissue diseases [86,87,88,89,90,91,92,93].

## 8. Mesotherapy

Mesotherapy is a cosmetic procedure involving the intradermal or subcutaneous injection of various substances, aimed at stimulating the regenerative processes of the skin and subcutaneous tissue. It was first performed in France by Pistor in 1952 [94]. The purpose of this treatment is to transport various active substances to the dermis in order to intensify collagenogenesis and fibroblast activity. Additionally, this procedure is designed to slow down elastin degeneration and transepidermal water loss [95]. In addition to the medical properties provided by injectable agents, the puncture of the skin itself stimulates proteins and genes that are activated in the wounding process, such as VEGF, β-catenin, Wnt3a, and Wnt3b [96]. In addition, agents, such as phosphatidylcholine, for dissolving adipose tissue, are used in mesotherapy cocktails to reduce subcutaneous fat deposits [97,98,99,100].

Data are scarce for the safety of mesotherapy in autoimmune diseases. Many substances used in mesotherapy do not undergo detailed controls, as in the case of drugs. They contain various mixtures, whose effects on the human body have not been fully tested. Although the authors did not identify any reports of an exacerbation of autoimmune thyroid disease after mesotherapy, the effect of such mixtures on patients with autoimmune thyroid diseases is difficult to assess.

The literature describes the sole use of thyroid hormones as ingredients in a mesotherapy cocktail for its lipolytic activity [101]. Such use, however, remains controversial, as thyrotoxicosis has been described following the use of a triiodothyronine-containing cocktail [102]. Chandrashekar et al. report on the positive effect of the use of steroids administered in mesotherapy in the therapy of alopecia areata [103], which is an autoimmune disease, unlike other authors who do not recommend mesotherapy in this disease [104]. On the other hand, there are reports of mesotherapy cocktails containing triiodothyroacetic acid, a thyroid hormone analogue indicated in the management of thyroid hormone resistance syndrome [105] that caused symptomatic thyrotoxicosis [106]. After cessation the therapy, thyroid functions returned to normal.

## 9. Conclusions

There is scarce information relating to the effectiveness and safety of aesthetic medicine in autoimmune thyroid disease. In a study by Reitblat et al. on a group of patients with various autoimmune diseases, such as rheumatoid arthritis, ankylosing spondylitis, and other systemic connective tissue diseases, through conducting noninvasive or minimally invasive cosmetic dermatologic procedures, the authors proved that these procedures are safe and do not cause autoimmune systemic disease exacerbation when performed in periods of remission [10]. Table 1 presents the effectiveness and safety of individual aesthetic medicine procedures in patients with autoimmune diseases of the thyroid gland, based on our analysis of the scientific literature. Out of the seven categories of treatments in aesthetic medicine analyzed by us, only two—mesotherapy and lifting threads—are not recommended due to the lack of safety studies and the potential possibility of a higher frequency of side effects in patients with autoimmune thyroid diseases.

## Figures and Tables

**Table 1:** Effectiveness and safety of aesthetic medicine procedures in patients with autoimmune diseases of the thyroid gland.

Procedure Type	Procedure Indications	Safety and Efficacy in Autoimmune Thyroiditis
Autologous fat grafting	Tissue volume restoration, facial contouring, facial asymmetry, scars, burns, radiation dermatitis, HIV-associated lipodystrophy, breast augmentation, buttock augmentation	Safe; however, graft survival may be reduced
Autologous platelet-rich plasma	Androgenic alopecia, acne scars, dermal augmentation (facial rhytides), therapies combined with fractional laser (resurfacing), autologous fat grafting (tissue augmentation), radio frequency (skin density)	Safe; however, efficacy may be reduced due to platelets disfunction, especially in hyperthyroidism.
Botulinum toxin	Wrinkle correction, strabismus, blepharospasm, chronic migraine	Safe and efficient
Hyaluronic acid	Tissue volume restoration, facial contouring, wrinkle correction, nonsurgical rhinoplasty	Relatively safe; however, inflammatory nodes may occur
IPL and laser treatment	Skin resurfacing, non-muscular wrinkles, telangiectatic naevi, acne rosacea, radiodermatitis, erythrosis of the neck, Ota’s nevus, Becker’s nevus, liver spots, eccrine angiomatous hamartoma, dermo-epidermal lesions	Safe and efficient
Lifting threads	face wrinkles, forehead lift, face lift	Unknown safety, procedure not recommended
Mesotherapy	Face wrinkles, insufficiently hydrated and nourished skin- skin rejuvenation, skin tightening, photoaging, alopecia, cellulite, local fat deposits, hyperpigmentation and melasma, telangiectasias, vitiligo, eczema	Unknown safety, procedure not recommended

## Data Availability

No additional data available.
